# Clinical Value the Neutrophil CD64 Index in Predicting the Severity of Hemorrhagic Fever With Renal Syndrome

**DOI:** 10.1002/iid3.70366

**Published:** 2026-04-05

**Authors:** Chuantao Ye, Rongrong Zhang, Shasha Wu, Min Wei, Jiaojiao Cao, Xinyi Du, Xiaofei Yang, Chao Fan, Bibo Kang, Jing Zhang, Xin Wei, Jianqi Lian

**Affiliations:** ^1^ Department of Infectious Diseases Second Affiliated Hospital of Air Force Medical University Xi'an Shaanxi China; ^2^ Department of Traditional Chinese Medicine First Affiliated Hospital of Air Force Military Medical University Xi'an Shaanxi China

**Keywords:** biomarker, hemorrhagic fever with renal syndrome, nCD64 index, PCT, SOFA

## Abstract

**Objective:**

The hemorrhagic fever with renal syndrome (HFRS) is an acute viral infectious disease characterized by multi‐system damage involving complex mechanisms. Effective identification of critically ill patients is essential for the prognosis of HFRS. The aim of this study was to investigate the utility of the neutrophil CD64 index (nCD64 index) in predicting the severity of HFRS.

**Methods:**

This study included 64 HFRS patients from the Center for Infectious Diseases of the Second Affiliated Hospital of Air Force Medical University between October 2023 and March 2024. The expression of CD64 on neutrophils was assessed using flow cytometry, and the nCD64 index was calculated. White blood cell (WBC), Procalcitonin (PCT), C‐reactive protein (CRP), Il‐6, and creatinine levels were measured for each patient, and Sequential Organ Failure Assessment (SOFA) scores were determined.

**Results:**

The expression of nCD64 was generally elevated in HFRS patients and demonstrated a positive correlation with WBC, neutrophil, lymphocyte, monocyte, and PCT, as well as a negative correlation with platelet. The receiver operating characteristic (ROC) curve of nCD64 index for predicting high SOFA score patients outperformed those of CRP, creatinine and WBC, and was equivalent to that of IL‐6 and PCT.

**Conclusion:**

The nCD64 index has the potential to serve as an effective biomarker for monitoring disease progression and assessing treatment efficacy in HFRS.

## Graphical abstract

(1) The expression of neutrophil CD64 index (nCD64 index) was generally elevated in hemorrhagic fever with renal syndrome patients and demonstrated a positive correlation with white blood cell (WBC), neutrophil, lymphocyte, monocyte, and procalcitonin (PCT), as well as a negative correlation with platelet. (2) The receiver operating characteristic curve of nCD64 index for predicting high sequential organ failure assessment score patients outperformed those of C‐reactive protein, creatinine and WBC, and was equivalent to that of IL‐6 and PCT.

## 1 Introduction

Hemorrhagic fever with renal syndrome (HFRS), caused by various serotypes of Hantaviruses within the family Hantaviridae of the order Bunyavirales, is widespread across the Eurasian continent [1, 2]. Globally, there are an estimated 60,000–150,000 cases of HFRS annually, with a majority occurring in China, accounting for 40%–50% of all cases [3]. The severity and outcome of HFRS depend on the specific serotype of Hantavirus involved. Infections with Hantaan viruses are notably associated with more severe disease [4].

Hantaviruses predominantly infect endothelial cells and induce a significant elevation in vascular endothelial permeability and inflammatory response. Laboratory findings commonly seen in HFRS include leukocytosis, thrombocytopenia, elevated levels of C‐reactive protein (CRP) and procalcitonin (PCT), which bear resemblance to bacterial sepsis. Acute thrombocytopenia is the hallmark of HFRS and serves as a significant factor in the diagnosis of HFRS [5]. Concurrently, in the absence of specific antiviral agents targeting hantavirus, the primary therapeutic approach relies on host immune‐mediated viral clearance and facilitation of vascular endothelial cell repair. Consequently, the quantity and functional status of both innate and adaptive immune cells (such as neutrophils, T cells, etc.) play a pivotal role in evaluating disease severity and prognostic prediction in hemorrhagic fever with renal syndrome (HFRS) [6].

Neutrophils are the predominant circulating leukocytes in humans and are widely recognized as one of the pivotal contributors to the acute inflammatory response. They're the first responder and the first line of defense against infection, especially in the early stage of bacterial infection [7]. Interestingly, a significant decrease in neutrophil levels is also observed during the early stages of HFRS, yet their precise role in the disease progression remains unclear [8]. The activation of neutrophils in HFRS may be linked to endothelial cell damage through the release of neutrophil extracellular traps (NETs), composed of extracellular chromatin adorned with histones and granular proteins such as myeloperoxidase (MPO) and human neutrophil elastase (HNE) [9, 10].

CD64, also known as Fc‐gamma receptor 1 (FcγR1), is predominantly expressed on the surface of antigen presenting cells such as macrophages, dendritic cells, and monocytes, with minimal expression on resting neutrophils [11, 12]. Following infection, particularly bacterial infection, there is a significant upregulation of CD64 on the surface of neutrophils within 1–6 h. Therefore, neutrophil (n)CD64 can serve as a promising biomarker for early detection of bacterial infections [13, 14]. however, its potential utility in virus‐mediated diseases such as HFRS remains understudied.

In this article, we aim to assess the expression of nCD64 index in HFRS patients. The nCD64 index was analyzed using flow cytometry and compared with conventional clinical indicators (WBC, PCT, and CRP) in high (≥ 6) and low (< 6) sequential organ failure assessment (SOFA) score groups through analysis of receiver‐operating characteristic curves (ROC), thus determining their clinical value in predicting disease severity.

## 2 Methods

### 2.1 Study Population

Between October 2023 and March 2024, a total of 64 patients diagnosed with HFRS sought assessment and treatment at the Center for Infectious Diseases of the Second Affiliated Hospital of Air Force Medical University. Upon admission, all enrolled patients tested positive for both Hantavirus‐specific IgM and IgG by colloidal gold assay. In addition, nine patients with bacterial infection were involved in this study.

### 2.2 Clinical Parameter Collection

A blood routine examination, along with the measurement of C‐reactive protein (CRP), procalcitonin (PCT), IL‐6, and creatinine, was conducted at the clinical laboratory of the Second Affiliated Hospital of Air Force Medical University. The SOFA score was calculated according to the Sepsis 3.0 criteria.

### 2.3 Quantification of the nCD64 Index

Blood samples were collected in EDTA anticoagulant tubes. Peripheral venous blood was obtained for the determination of neutrophil CD64 expression using flow cytometry (BD FACSAria II, BD, USA). One hundred microliters of peripheral blood was incubated with 20 μL CD14‐FITC, CD45‐PerCP and CD64‐PE monoclonal fluorescent antibody (BD, USA) for 15 min in the dark after gentle vortexing. Subsequently, FACSLysin (1 mL) was added and incubated at room temperature away from direct light for 10 min. The cells were then analyzed using flow cytometry to measure the mean fluorescence intensity of lymphocytes, monocytes, and neutrophils. Based on these measurements, the nCD64 index was calculated as: (neutrophils–lymphocytes)/(monocytes–neutrophils).

### 2.4 Statistical Analysis

The comparison of two sets of measurement data utilized an independent sample *t*‐test. Receiver operator characteristics (ROC) curve was constructed to assess the effectiveness of various clinical characteristics in predicting higher SOFA score. Statistical significance was determined if the results showed *p* < 0.05.

## 3 Results

### 3.1 Basic Clinical Characteristics of the Study Population

A total of 63 patients with HFRS and 19 patients with bacterial infection patients were involved in this study. The clinical and laboratory characteristics of all patients were presented in Table 1. There was no significant difference in age and gender among them. However, HFRS patients had a higher SOFA score than bacterial infection patients', but with no significant difference in inpatient days and mortality.

**Table 1:** Baseline characteristics of enrolled patients.

Characteristics	HFRS (*N* = 63)	Bacterial infection (*N* = 19)	*p* value
Age (years)	45.81 (17.37)	54.5 (17.6)	0.0627
Male	50 (79.37%)	10 (52.63%)	0.4365
SOFA score	6.92 (5.42)	1.74 (4.47)	0.0003
Inpatient days	15.26 (11.86)	13.63 (6.32)	0.5728
Mortality, *n* (%)	3 (4.76%)	3 (15.79%)	0.5000
WBC (10^9^/L)	12.67 (11.50)	7.96 (3.67)	0.0862
Neutrophil (10^9^/L)	7.83 (7.42)	5.65 (3.23)	0.2220
Monocyte (10^9^/L)	1.61 (2.00)	0.57 (0.31)	0.0292
Lymphocyte (10^9/L)	3.07 (3.06)	1.56 (1.33)	0.0431
Platelet (10^9^/L)	156.24 (137.01)	202 (121)	0.2002
PCT (ng/mL)	3.34 (6.62)	2.90 (8.39)	0.8137
CRP (mg/L)	21.69 (18.05)	30.50 (27.48)	0.1192
IL‐6 (pg/mL)	46.13 (72.50)	89.05 (234.33)	0.2397
Creatinine (μmol/L)	276.13 (133.12)	89.61 (95.45)	< 0.0001
nCD64 index	4.34 (5.15)	4.16 (5.51)	0.8988
HLA‐DR	95.53 (6.24)	87.99 (12.79)	0.0009

### 3.2 nCD64 Index Increased in HFRS Patients

**Figure 1:**
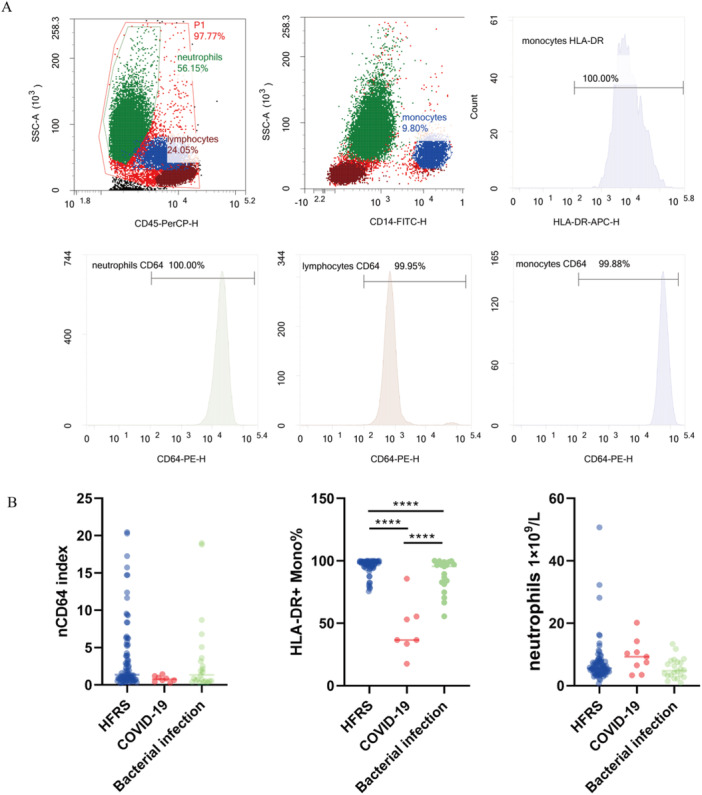
Flow cytometric analysis of CD64 and HLA‐DR expression. (A) Nucleated cells were gated according to SSC and pan‐leucocyte marker CD45‐PerCP distribution. Lymphocytes (brown), neutrophils (green), and monocytes (blue) were gated from nucleated cells according to CD14 distribution. The histogram of CD64 expression (MFI) on neutrophils, lymphocytes, and monocytes and HLA‐DR expression of monocytes were calculated. (B) The nCD64 index showed no significant difference among HFRS, COVID‐19 and bacterial infection patients.

Due to significantly increases in bacteria‐infected diseases, nCD64 index was considered as a candidate indicator for diagnosing, monitoring bacterial infection, and evaluating antibiotic therapy [15, 16]. To describe the profile of nCD64 index among HFRS, nCD64 index of 83 samples from 63 patients were measured using flow cytometry (Figure 1A). The results shown that 53 (63.86%) samples had abnormal nCD64 index, and shown no significant difference compared with nCD64 index of bacterial infection patients (Figure 1B). Certainly, the median neutrophil count in COVID‐19 patients was marginally elevated compared to that observed in HFRS and bacterial infection patients (nCD64 index among COVID‐19 were derived from our previously unpublished data). However, intriguingly, the nCD64 index in COVID‐19 patients was only slightly attenuated in comparison to the indices found in HFRS and bacterial infection patients. This nuanced difference underscores the complex interplay of immune markers in these distinct disease states.

### 3.3 No Significant Down‐Regulation of HLA‐DR on Monocytes in HFRS Patients

The diminished expression of human leukocyte antigen class II (HLA‐DR) on monocytes stands as a pivotal indicator of immune system malfunction in critically ill individuals, potentially serving as a valuable companion diagnostic tool and clinical biomarker, alongside the nCD64 index, for assessing the severity of infection [17, 18]. To elucidate this phenomenon, flow cytometry was employed to quantify HLA‐DR expression levels on monocytes across patient cohorts with HFRS, COVID‐19, and bacterial infections. Notably, a stark reduction in HLA‐DR expression was observed in monocytes from COVID‐19 and bacterial infection patients compared to those with HFRS, underscoring the absence of significant immune dysfunction in HFRS patients (Figure 1A).

### 3.4 Correlation Analysis Between nCD64 Index and Basic Clinical Characteristics

**Figure 2:**
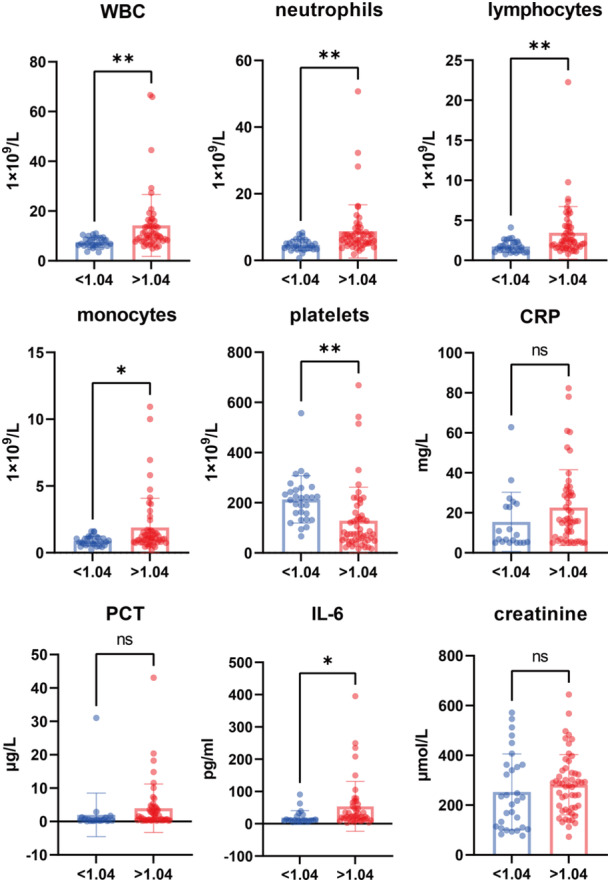
WBC, neutrophil, lymphocyte, monocyte and IL‐6 increased significantly in HFRS patients with higher nCD64 index, while platelet declined markedly. There had no significance difference on CRP, PCT, and creatinine.

**Figure 3:**
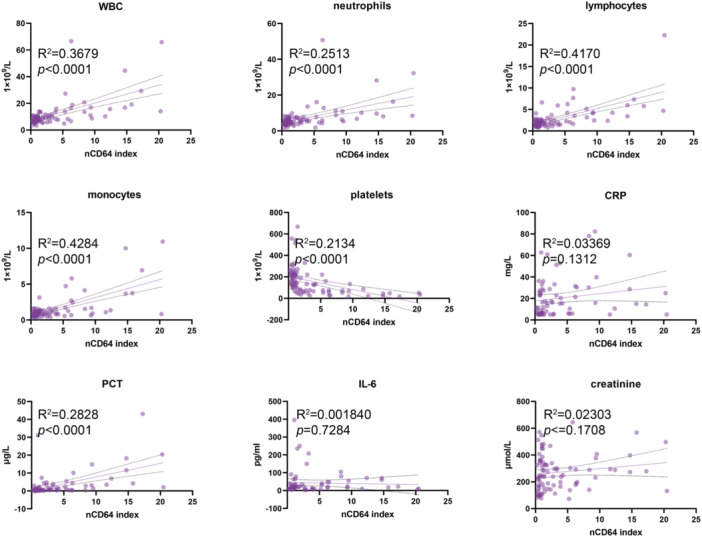
The CD64 index had a positive correlation with WBC, neutrophil, lymphocyte, monocyte and PCT, and a negative correlation with platelet in HFRS.

It is observed that some sepsis associated clinical characteristics like WBC, PCT, CRP and IL‐6 could increase after Hantaviruses infection [19]. Based on the mean value of nCD64 index, HFRS patients were divided into two groups, normal and higher nCD64 index group. We want to know the difference between HFRS patients with normal and higher nCD64 index in the level of WBC, PCT, CRP, IL‐6, and other clinical characteristics. The results shown that WBC, neutrophil, lymphocyte, monocyte and IL‐6 increased significantly in HFRS patients with higher nCD64 index, while platelet declined markedly. There had no significance difference on CRP, PCT and creatinine (Figure 2). Further, the correlation between nCD64 index and WBC, PCT, CRP, IL‐6, and other clinical characteristics was analyzed. The results indicated a significant positive correlation between nCD64 index with WBC, neutrophil, lymphocyte, monocyte and PCT, and a negative correlation with platelet, whereas others have shown no meaningful correlation (Figure 3).

### 3.5 Prognostic Value of nCD64, PCT, CRP, WBC Levels in HFRS

**Figure 4:**
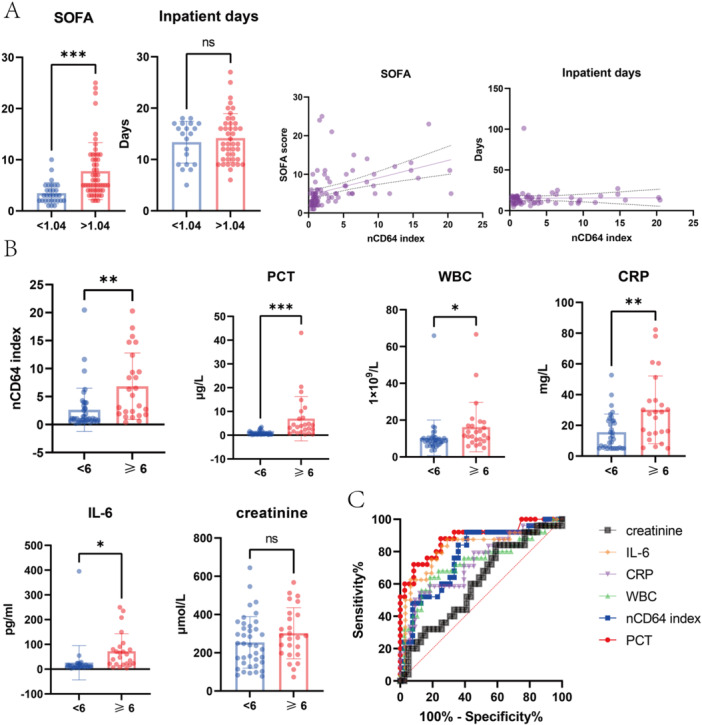
Prognostic value of nCD64, PCT, CRP, WBC levels in HFRS. (A) HFRS patients with higher nCD64 index tend to have higher SOFA score and there was a positive correlation between nCD64 index and SOFA score. (B) There was a markedly elevated nCD64 index, WBC, PCT, CRP, IL‐6 in HFRS patients with ≥ 6 SOFA score compared ones with < 6 SOFA score. (C) ROC curves for the SOFA based severity assessment of HFRS.

SOFA score was developed to assess the acute morbidity of critical illness at a population level and has been widely validated as a tool for this purpose across a range of healthcare settings and environments [20, 21]. HFRS patients with higher nCD64 index tend to have higher SOFA score and there was a positive correlation between nCD64 index and SOFA score, but not with inpatient days (Figure 4A). To further test the performance of nCD64 index in the severity assessment of HFRS, all patients were divided into two groups: high SOFA score (≥ 6) (*n* = 25; 20 males and 5 females) and lower SOFA score (< 6) (*n* = 39; 31 males and 8 females) based on the average SOFA score (6.83 ± 5.43). Patients with ≥ 6 SOFA score showed a significant increase in nCD64 index compared with ones with< 6 SOFA score (6.846 vs 2.632, *p *< 0.001) (Figure 4B). ROC curve analysis established 1.18 as the best cut‐off value of nCD64 index to identify patients with ≥ 6 SOFA score, with a sensitivity of 92% (95% confidence interval [CI], 75.03%–98.58%) and specificity of 58.97% (95% CI, 43.42%–72.92%) (Figure 4C). Further, the area under roc curve (AUC) of PCT (area = 0.8800, *p* < 0.0001) and IL‐6 (area = 0.8172, *p* < 0.0001) were higher than that of nCD64 index (area = 0.7687, *p* = 0.0003), the AUC of CRP (area = 0.7323, *p* = 0.0029) and WBC (area = 0.7284, *p* = 0.0023) were less than that of nCD64 index. The AUC of creatinine (area = 0.6056, *p* = 0.1564) was the smallest and had no statistical significance (Figure 4C).

## 4 Discussion

In addition to the direct damage caused by viral infection of vascular endothelial cells, the excessive inflammatory response is considered to be the primary pathogenic mechanism of HFRS [22]. During the early stages, various immune cells such as monocytes, macrophages, B‐cells, neutrophils, and CD8+ (cytotoxic) T‐cells are activated [23, 24, 25]. However, little was known about the role and phenotype of neutrophils. This study revealed a significant increase in CD64 expression in neutrophils of HFRS patients. Given the role of CD64 as a high‐affinity type I immunoglobulin Fc receptor (FcγR1) for recognizing IgG1 and lgG3, it is suggested that Antibody‐dependent cellular cytotoxicity (ADCC) may be an efficient way for activating neutrophils and involved in eliminating Hantaviruses.

A growing body of research indicates that the nCD64 index can serve as a biomarker for diagnosing, monitoring, and evaluating the prognosis of bacterial infections However, its role in viral infections remains unclear. It has been reported that the nCD64 index was utilized to distinguish between bacterial and viral infections in COVID‐19 patients, showing significant differences between survivors and nonsurvivors [26]. In our study on patients with HFRS, we observed an increase in the nCD64 index among those with higher SOFA scores. Furthermore, correlation analysis and ROC analysis demonstrated that the nCD64 index could effectively indicate the severity and progression of HFRS. Additionally, it showed greater predictive efficacy than CRP, IL‐6, WBC, and creatinine.

The upregulation of CD64 expression on neutrophils occurs rapidly within 4–6 h of infection, induced by interferon‐γ (IFN‐γ), granulocyte monocyte colony stimulating factor (GM‐CSF) and other cytokines [27]. IFNs‐mediated antiviral responses play a central role in the host defense against viral infection. It has been reported that serum levels of IFN‐γ are elevated in the early stages of HFRS and can reduce HTNV replication [28]. We hypothesize that the elevation, activation, and expression of CD64 on neutrophils may represent another important antiviral mechanism mediated by IFNs.

Decreased HLA‐DR expression in monocytes levels have been described as an indicator of immunosuppression, mainly mediated by glucocorticoids and IL‐10 [29]. It was revealed that TNF‐α, IL‐6, IFN‐γ, and IL‐8 are main cytokines that was elevated during HFRS. And glucocorticoids were used in small amounts and briefly in the febrile phase of HFRS, which was not routinely used in all stages of HFRS. Thus, no significant reduction of HLA‐DR expression in HFRS was observed. And further confirmed that immune pathological damage is the main damaging mechanism of HFRS [30].

Despite the strengths possessed by the present study, there exist certain limitations. First, it is important to note that this is a single center study, which may limit the generalizability of our findings. Second, all participants in this study were Chinese inpatients with HFRS, so further investigation is needed to determine the relationship between HFRS severity and complex inflammatory markers in non‐Chinese populations. Third, the nCD64 index was not tested continuously. In the forthcoming days, we will further probe into the dynamic alterations and mechanism of increased expression of nCD64 in HFRS patients. Meanwhile, given the involvement of neutrophils in inflammatory responses, vascular endothelial cell injury, and viral clearance processes, the correlation between nCD64 and VEGF, thrombomodulin, TNF‐α, as well as serum viral load warrants further investigation.

In conclusion, our study has, for the first time, demonstrated elevated nCD64 expression in HFRS patients and its positive correlation with white blood cell (WBC) count, neutrophils, lymphocytes, monocytes, and procalcitonin (PCT), as well as a negative correlation with platelet count. Furthermore, the nCD64 index exhibits superior predictive value for the prognosis of HFRS patients with higher Sequential Organ Failure Assessment (SOFA) scores compared to C‐reactive protein (CRP), creatinine, and WBC. Therefore, the nCD64 index may serve as a promising biomarker for monitoring disease progression and assessing treatment efficacy in HFRS.

## Author Contributions

**Chuantao Ye:** data analysis, drafting of the manuscript. **Rongrong Zhang:** flow cytometry. **Shasha Wu:** clinical data statistics. **Min Wei:** clinical data collection. **Jiaojiao Cao:** clinical data collection. **Xinyi Du:** clinical data collection. **Xiaofei Yang:** data analysis and result interpretation. **Chao Fan:** paper review. **Bibo Kang:** data analysis and result interpretation. **Jing Zhang:** guidance of experiment. **Xin Wei:** guidance of experiment. **Jianqi Lian:** guidance of experiment. All authors read and approved the final version.

## Ethics Statement

The study was approved by the Institutional Review Board and Medical Ethics Committee of the second affiliated hospital of the air force military medicine (No.: 201912‐07).

## Conflicts of Interest

The authors declare no conflicts of interest.

## Data Availability

The authors have nothing to report.
